# Correlation between Urothelial Differentiation and Sensory Proteins P2X3, P2X5, TRPV1, and TRPV4 in Normal Urothelium and Papillary Carcinoma of Human Bladder

**DOI:** 10.1155/2014/805236

**Published:** 2014-04-27

**Authors:** Igor Sterle, Daša Zupančič, Rok Romih

**Affiliations:** ^1^Department of Urology, University Medical Centre Ljubljana, Zaloška cesta 2, 1000 Ljubljana, Slovenia; ^2^Institute of Cell Biology, Faculty of Medicine, University of Ljubljana, Vrazov trg 2, 1000 Ljubljana, Slovenia

## Abstract

Terminal differentiation of urothelium is a prerequisite for blood-urine barrier formation and enables normal sensory function of the urinary bladder. In this study, urothelial differentiation of normal human urothelium and of low and high grade papillary urothelial carcinomas was correlated with the expression and localization of purinergic receptors (P2X3, and P2X5) and transient receptor potential vanilloid channels (TRPV1, and TRPV4). Western blotting and immunofluorescence of uroplakins together with scanning electron microscopy of urothelial apical surface demonstrated terminal differentiation of normal urothelium, partial differentiation of low grade carcinoma, and poor differentiation of high grade carcinoma. P2X3 was expressed in normal urothelium as well as in low grade carcinoma and in both cases immunolabeling was stronger in the superficial cells. P2X3 expression decreased in high grade carcinoma. P2X5 expression was detected in normal urothelium and in high grade carcinoma, while in low grade carcinoma its expression was diminished. The expression of TRPV1 decreased in low grade and even more in high grade carcinoma when compared with normal urothelium, while TRPV4 expression was unchanged in all samples. Our results suggest that sensory proteins P2X3 and TRPV1 are in correlation with urothelial differentiation, while P2X5 and TRPV4 have unique expression patterns.

## 1. Introduction

The urothelium, which lines the urinary bladder, performs two major functions. The first one is a well-characterized high resistance permeability barrier, and the second, not so well understood, is a sensory function. Permeability barrier is formed and maintained during urothelial differentiation, which reaches the terminal stage in superficial umbrella cells. Umbrella cells synthesize four major transmembrane proteins, uroplakins (UPIa, UPIb, UPII, and UPIIIa), which form unique membrane specialization, that is, urothelial plaques [1]. It was shown that uroplakins directly contribute to the urothelial barrier function [2]. After synthesis and modifications of uroplakins in the endoplasmic reticulum and the Golgi apparatus, respectively, urothelial plaques are gradually assembled in post-Golgi compartments. They are transported to the apical plasma membrane of umbrella cells by fusiform vesicles [3–5]. Urothelial plaques are encircled by so-called hinge regions, which form microridges at the urothelial apical surface [6].

Urothelium, together with lamina propria, acts also as a sensory web, which is able to receive, amplify, and transmit information about its environment [7]. Numerous receptors and ion channels, including purinergic P2X receptors and transient receptor potential vanilloid (TRPV) channels, have been identified in urothelial cells. They respond to bladder filling, changes of urine composition, or autocrine and paracrine mediators [8]. P2X receptors and TRPV channels are relatively nonselective cation channels [9, 10]. Stretching triggers chemically mediated activation of purinergic P2X receptors and exocytosis of fusiform vesicles [11]. Moreover, stretching stimulates afferent nerve processes and may signal the degree of bladder filling to the central nervous system [12, 13]. TRPV channels may also be involved in response to mechanical and chemical stimuli [14, 15]. It has been proposed that TRPV1 and TRPV4 are involved in bladder filling sensation and regulation of the voiding reflex [16–18].

We have shown previously that uroplakin expression decreases during bladder carcinogenesis [19–21], which is reflected in partial urothelial differentiation and barrier disruption [22, 23]. Compromised permeability barrier results in lower urinary tract symptoms (LUTS), which are divided into three categories: storage, voiding, and postmicturition symptoms. Storage symptoms include increased micturition frequency, nocturia, urinary urgency, and urinary incontinence [24]. Common voiding symptoms contain slow or weak stream, hesitancy, and terminal dribble. Postmicturition symptoms include the sensation of incomplete emptying and postmicturition dribble [24, 25]. Although the aetiology of LUTS is multifactorial, bladder carcinomas represent one of the possible causes [26]. Since P2X receptors are implicated in the bladder sensation mediated by afferent nerves, it is likely that sensory web plays an important role in some bladder diseases accompanied by LUTS [27]. Currently, very little is known about the roles of P2X receptors and TRPV channels in human bladder tumours. It is assumed that P2X receptors, activated by ATP, have a significant antineoplastic action and might be involved in urothelial differentiation in high grade superficial bladder cancer [28]. Regarding TRPV1, its downregulation was reported in superficial and muscle invasive urothelial cancers [17, 29].

Here we report on the expression and localization of P2X3, P2X5, TRPV1, and TRPV4 in normal human urothelium and in low and high grade papillary urothelial carcinomas. The results are compared with uroplakin expression and urothelial apical surface ultrastructure. Our results suggest correlation between sensory function of the urothelium and urothelial differentiation.

## 2. Material and Methods

### 2.1. Patients and Sampling

The study was conducted in accordance with the Helsinki Declaration and approved by the Slovenian National Medical Ethics Committee number 76/10/10. Eighteen patients with papillary urothelial carcinoma who underwent transurethral resection of the bladder were included in the study. Informed consent was obtained from all patients. Two samples were acquired by cold-cup biopsies from each patient: (i) the urothelial tumour and (ii) the normal urothelium 1 cm posterior from the interureteric ridge. Biopsies captured urothelium and lamina propria. For pathological staging and grading, EAU Guidelines on Non-muscle-invasive Bladder Cancer [30] were used. Urothelial tumours were diagnosed as low grade papillary urothelial carcinoma with no lamina propria invasion: pTa (12 patients; 60 to 88 years old; mean age 73.2 years), high grade papillary urothelial carcinoma with lamina propria invasion: pT1 or with muscularis propria invasion: pT2 (6 patients; 57 to 70 years old; mean age 65.5 years). Regarding normal samples, only those showing no signs of hyperplasia or dysplasia were further processed (13 samples). Each sample was processed for Western blotting, immunofluorescence, and scanning electron microscopy.

### 2.2. Western Blotting

Samples were homogenized in ice-cold buffer (0.8 M Tris-HCl, 7.5% SDS, and 1 mM phenylmethylsulfonyl fluoride). The lysates were centrifuged and the protein concentration in the supernatant was determined by using a BCA protein assay kit (Pierce, Rockford, IL). From each patient, the protein sample (50 *μ*g/lane) from the normal urothelium was loaded next to the protein sample (50 *μ*g/lane) from the urothelial tumour. Proteins were size fractionated on 7.5%, 10%, or 12% SDS-polyacrylamide gels and then transferred to Hybond ECL nitrocellulose membranes (Amersham Biosciences, Buckinghamshire, UK) by electroblotting. After blocking overnight at 4°C in 5% skim milk in phosphate buffer saline with 0.1% Tween (PBS-Tween), membranes were incubated for 2 hours at room temperature with rabbit polyclonal anti-uroplakin (1 : 10.000; kindly provided by Tung-Tien Sun, New York University Medical School, USA), guinea pig polyclonal anti-P2X3 (1 : 1000; cat. number NB100-1658, Novus Biologicals, Littleton, USA), goat polyclonal anti-P2X5 (1 : 200; cat. number sc-15192, Santa Cruz Biotechnology, Dallas, USA), rabbit polyclonal anti-TRPV1 (1 : 200; cat. number ACC-030, Alomone Labs, Jerusalem, Israel), or rabbit polyclonal anti-TRPV4 (1 : 500; cat. number ab39260, Abcam, Cambridge, UK). After washing in PBS-Tween, membranes were incubated for 1 hour, depending on primary antibody either with horseradish peroxidase-conjugated goat anti-rabbit (1 : 5000; Santa Cruz Biotechnology), goat anti-guinea pig (1 : 5000; Santa Cruz Biotechnology), or donkey anti-goat (1 : 7000; Jackson ImmunoResearch Laboratories, West Baltimore Pike, USA). Membranes were finally probed with enhanced chemiluminescence reagent (ECL; Amersham Biosciences, Buckinghamshire, UK) and exposed to X-ray films. To confirm equal protein loading, the blots were stripped with Restore Western Blot Stripping Buffer (Pierce, Rockford, IL) and reprobed with anti-actin antibody (diluted 1 : 2000; Sigma, Taufkirchen, Germany).

### 2.3. Immunofluorescence

Samples were fixed with 4% formaldehyde in PBS for 2.5 hours at 4°C. They were washed and impregnated with 30% sucrose, embedded in OCT mounting medium (Tissue Tek, Sakura Finetek Europe B.V., The Netherlands), and frozen in liquid nitrogen. Frozen sections were cut in cryostat at −25°C, collected on glass slides, and air dried. Sections were incubated in 1% BSA in PBS for 1 hour. Immunolabeling was performed using the same antibodies as for Western blotting: antibodies against uroplakins (1 : 10.000), P2X3 (1 : 1000), P2X5 (1 : 200), TRPV1 (1 : 200), and TRPV4 (1 : 500). After washing with PBS, sections were incubated for 90 minutes either with rabbit anti-goat AlexaFluor555 for uroplakins (Molecular Probes), donkey anti-guinea pig TRITC or FITC for P2X3 (Jackson ImmunoResearch Europe Ltd.), rabbit anti-goat AlexaFluor555 for P2X5 (Invitrogen), or goat anti-rabbit AlexaFluor488 for TRPV1 and TRPV4 (Molecular Probes). All secondary antibodies were diluted 1 : 400 in 0.1% bovine serum albumin (BSA) in PBS. A series of negative controls were performed, omitting the primary antibody or incubating sections with nonrelevant antibodies. Sections were washed, stained with DAPI, and immersed in Vectashield embedding medium. Slides were examined with a fluorescence microscope Eclipse TE300 (Nikon).

### 2.4. Scanning Electron Microscopy

Samples were fixed in 4.5% paraformaldehyde and 2% glutaraldehyde for 3 hours. The samples were postfixed in osmium tetroxide, dehydrated in ethanol, and critical-point dried. After sputter-coating with gold, they were examined at 15 kV with a Jeol JSM 840A scanning electron microscope (Jeol Ltd., Tokyo, Japan).

## 3. Results

### 3.1. Normal Urothelium

**Figure 1:**
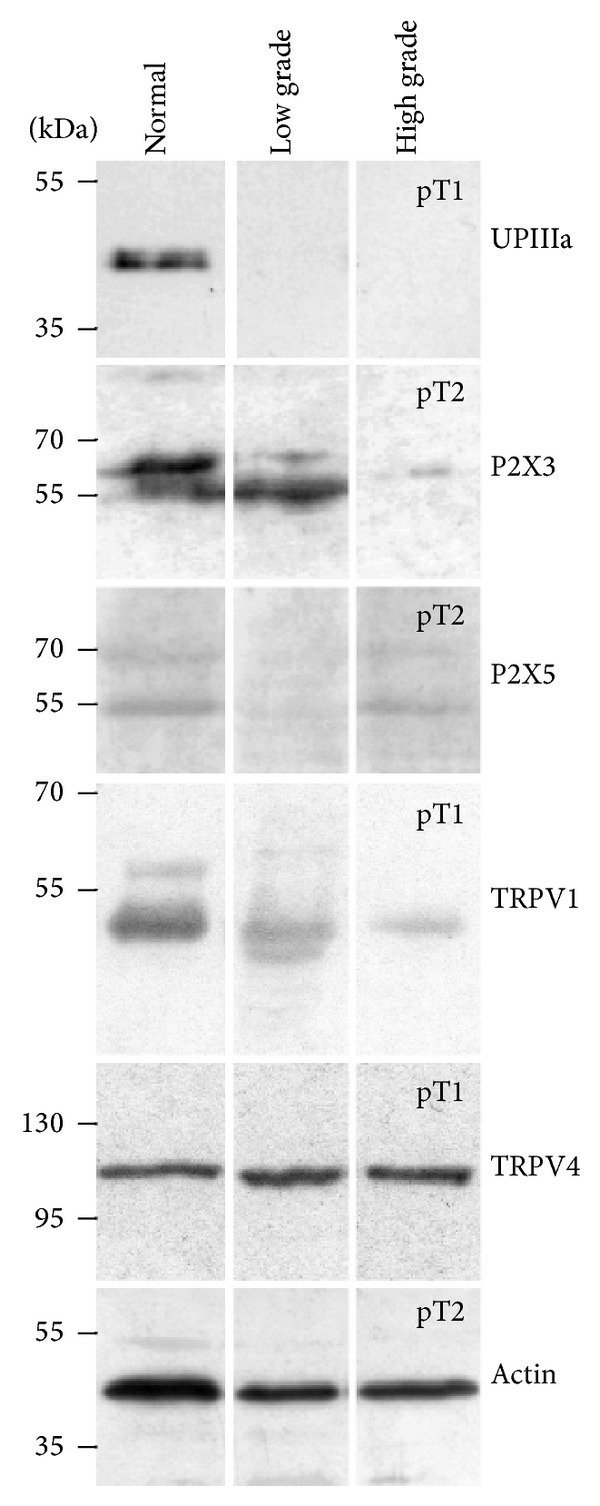
The expression pattern of uroplakins, P2X3, P2X5, TRPV1, and TRPV4 in normal human urothelium and in low and high grade papillary urothelial carcinomas as determined by Western blotting. In the protein samples of normal urothelium, UPIIIa, P2X3, P2X5, TRPV1, and TRPV4 are expressed. In low grade carcinoma, there is no expression of uroplakins. P2X3 and TRPV4 are expressed as in normal urothelium, while P2X5 is greatly diminished. TRPV1 expression is decreased in comparison to normal urothelium. In the protein samples of high grade carcinoma with lamina propria invasion (pT1) and those with muscularis propria invasion (pT2), the expression patterns were similar and therefore three examples of each are presented here. The expression of uroplakins is negative and expressions of P2X3 and TRPV1 are substantially decreased compared to normal urothelium. The expressions of P2X5 and TRPV4 are the same as in normal urothelium. Western blots were done in duplicate. Molecular weights are shown in kilodaltons (kDa).

**Figure 2:**
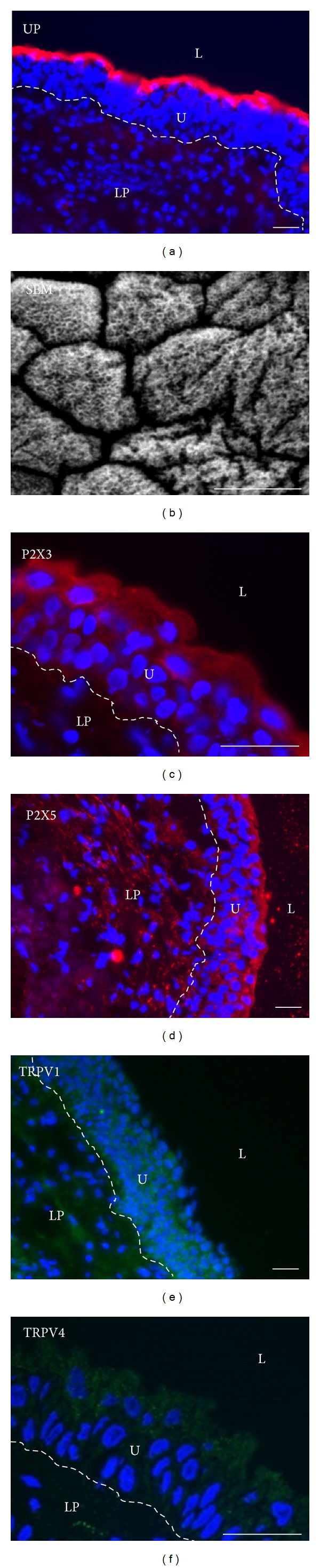
Normal human urothelium (U). (a) Strong uroplakin (UP) immunolabeling (red) is restricted to umbrella cells. (b) Scanning electron microscopy (SEM) shows large polygonal umbrella cells with microridges on the urothelial apical surface. (c) Antibodies against P2X3 and (d) P2X5 label (red) all layers of the normal urothelium. The reaction is stronger in umbrella cells than in basal cells. (e) Anti-TRPV1 antibody labels (green) all urothelial cell layers. (f) Anti-TRPV4 labelling (green) is weak in basal and intermediate cell layers and moderate in umbrella cells. In (a), and (c)–(f) nuclei are labelled blue with DAPI. L = lumen, LP = lamina propria. Scale bars = 50 *μ*m.

Uroplakins, differentiation dependent and urothelium-specific transmembrane proteins, were detected by polyclonal anti-uroplakin antibody, which reacts strongly with UPIIIa (47 kDa). The expression of UPIIIa in all samples of normal human urothelium was positive (Figure 1). To localize uroplakins within urothelium we used immunofluorescence. Normal urothelium showed strong uroplakin labelling of the superficial cells (Figure 2(a)). Scanning electron microscopy revealed large, polygonal cells covering urothelial surface (Figure 2(b)). They had scalloped appearance with microridges, demonstrating the presence of urothelial plaques. All features indicated above provided evidence that umbrella cells formed the superficial cell layer of normal urothelium, and they were therefore considered to be terminally differentiated. Confirming this, we further analysed the expression and distribution of four nonselective ion channels.

Western blotting confirmed the expression of P2X3 (approximately 60 kDa) in the normal urothelium (Figure 1). Antibodies against P2X3 labelled all urothelial cell layers in all samples of normal urothelium, with more intense labelling in the superficial layer, where umbrella cells are located (Figure 2(c)). Regarding P2X5, the expression of monomer protein (70 kDa) was confirmed, while dimmer protein (140 kDa) was not detected by Western blotting (Figure 1). An additional band of approximately 50 kDa was also observed with anti-P2X5 antibody. In immunofluorescence, anti-P2X5 antibody labelled all urothelial cell layers in all samples of normal urothelium, with weaker labelling intensity in the basal than in the superficial layer (Figure 2(d)).

Data on TRPV1 molecular weight range from 30 to 150 kDa. Anti-TRPV1 antibody, which was used in this study, revealed the most intense band for TRPV1 at approximately 50 kDa and weak band at approximately 60 kDa (Figure 1). Immunolabeling of TRPV1 was evident in all urothelial cell layers in all samples of normal urothelium (Figure 2(e)). Anti-TRPV4 antibody used in this study detected band at 104 kDa, as predicted. TRPV4 immunolabeling was weak in basal and intermediate cell layers and moderate in umbrella cells in all samples of normal urothelium (Figure 2(f)).

### 3.2. Low Grade Papillary Urothelial Carcinoma

**Figure 3:**
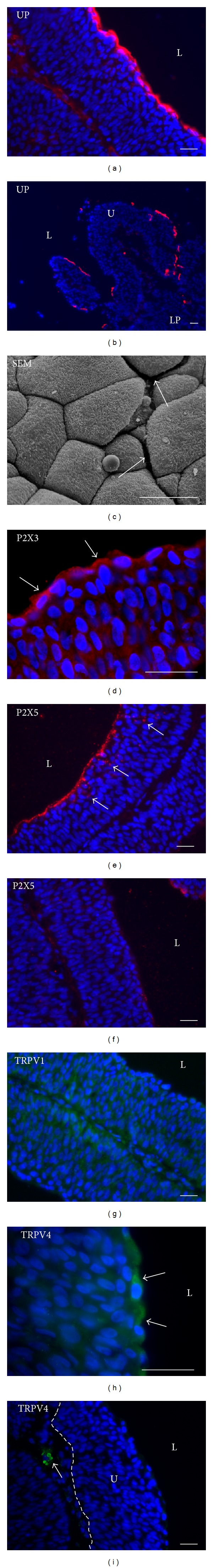
Low grade papillary urothelial carcinoma (pTa). (a) Uroplakin (UP) immunolabeling (red) is detected either continuously throughout the urothelial (U) superficial cell layer or (b) as regions where some superficial cells are uroplakin positive (red) and some uroplakin negative. (c) Scanning electron microscopy (SEM) reveals altered appearance of the urothelial apical surface in comparison to normal urothelium. Some neighbouring superficial cells are separated from one another (arrows) and underlying intermediate cell can be seen (asterisk). (d) P2X3 immunolabeling (red) is positive in all urothelial cell layers with the strongest immunolabeling in the superficial cells (arrows). (e) Some regions are intensely immunolabeled with anti-P2X5 antibody (red) in the urothelial superficial cell layer and in individual intermediate cells (arrows), (f) while other regions are P2X5 negative. (g) TRPV1 immunolabeling (green) is seen in basal and intermediate cells, but not in superficial cells. (h) In some regions, superficial cells (arrows) are TRPV4 positive (green), while (i) in other regions all urothelial cells are TRPV4 negative. TRPV4 positive immunolabeling is seen in the compartments of the lamina propria (arrow). In images (a)-(b) and (d)–(I), nuclei are labelled blue with DAPI. L = lumen, LP = lamina propria. Scale bars = 50 *μ*m.

In low grade papillary urothelial carcinoma, decreased or abolished uroplakin expression was detected (Figure 1) when compared to normal urothelium. In all samples of low grade papillary urothelial carcinoma, immunolabeling of uroplakins was heterogeneous and we were able to discriminate (i) regions with uroplakin positive labelling of all superficial cells (Figure 3(a)), (ii) regions with uroplakin positive and uroplakin negative superficial cells (Figure 3(b)), and (iii) regions with only uroplakin negative superficial cells. Urothelial apical surface displayed altered appearance in comparison to normal urothelium with disrupted superficial cell layer and gaps between adjacent superficial cells (Figure 2(c)). Superficial cells were not covered with microridges and they were smaller in comparison to umbrella cells. Taken together, these results indicated partial differentiation of superficial cells in low grade carcinoma.

In the protein samples of low grade carcinoma strong expression of P2X3 was detected (Figure 1). In all samples of low grade papillary urothelial carcinoma, P2X3 immunolabeling was similar as in normal urothelium; that is, the labelling of the superficial cell layer was the strongest (Figure 3(d)). Western blotting revealed that P2X5 expression was much weaker in the low grade carcinoma than in normal urothelium (Figure 1). By P2X5 immunolabeling, two types of regions were observed in all samples: (i) regions with immunolabeling of superficial cells and individual intermediate cells (Figure 3(e)) and (ii) regions with negative immunolabeling in all cell layers, except weak labelling in individual superficial cells (Figure 3(f)).

TRPV1 expression determined by Western blotting was weaker in the low grade carcinoma than in normal urothelium (Figure 1). Immunofluorescence of TRPV1 revealed stronger immunolabeling in the basal and in the intermediate cell layers than in the superficial cell layer (Figure 3(g)). In the low grade carcinoma, TRPV4 expression was stronger or equal to the expression of TRPV4 in the normal urothelium (Figure 1). Regarding TRPV4 immunolabeling, two types of regions were discriminated in all samples: (i) regions with positive immunolabeling of superficial and intermediate cell layers (Figure 3(h)) and (ii) regions with negative reaction in all urothelial cell layers (Figure 3(i)). Positive TRPV4 immunolabeling was seen in the lamina propria.

### 3.3. High Grade Papillary Urothelial Carcinoma

**Figure 4:**
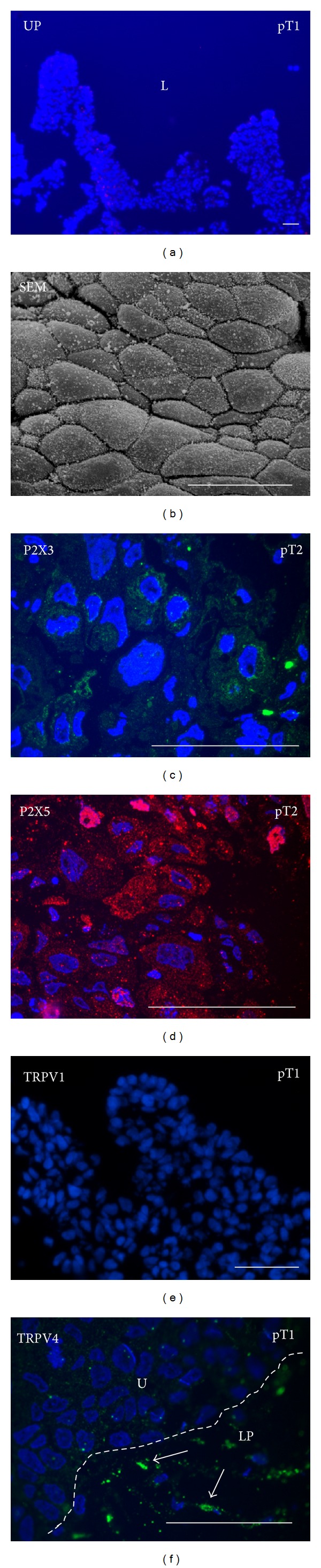
High grade papillary urothelial carcinomas (pT1 or pT2). (a) Uroplakin (UP) labelling (red) is negative. (b) Scanning electron microscopy (SEM) shows that superficial urothelial cells are small and polymorphic. They have microvilli on their apical surface. (c) Antibody against P2X3 weakly labels (green) urothelial cells. (d) P2X5 labelling (red) is present in all urothelial cells. Nuclei of some cells are also labelled. (e) TRPV1 labelling is negative. (f) Weak labelling of TRPV4 in urothelial cells (U) is seen, but strong TRPV4 labelling (arrows) is seen in the compartments of the lamina propria (LP). In images (a) and (c)–(f), nuclei are labelled blue with DAPI. L = lumen. Scale bars = 50 *μ*m.

We did not observe any difference between pT1 and pT2 high grade papillary urothelial carcinoma with respect to the protein expression and localization studied here. Western blotting showed that there was no uroplakin expression in these samples (Figure 1) and uroplakin immunolabeling was also negative in all samples of pT1 and pT2 (Figure 4(a)). Scanning electron microscopy revealed superficial cells of different sizes, but prevailing ones were smaller than in normal urothelium (Figure 4(b)). They were covered with microvilli (Figure 4(b)), which are found only on poorly differentiated superficial urothelial cells [6].

The expression of P2X3 was greatly decreased in all samples of the high grade carcinoma when compared to normal urothelium or to low grade carcinoma (Figure 1). In all samples of high grade carcinoma, antibodies against P2X3 weakly labelled all cell layers (Figure 4(c)). Western blotting confirmed the expression of monomer form of P2X5, which was similar in pT1 and pT2 protein samples (Figure 1). As observed by Western blotting, the expression of P2X5 was higher in high grade than in low grade carcinoma. P2X5 was labelled in all cell layers of all pT1 and pT2 samples (Figure 4(d)).

By Western blotting, the expression of TRPV1 was the lowest in high grade carcinoma in comparison to normal urothelium and to low grade carcinoma (Figure 1). By immunofluorescence, all samples of high grade carcinoma were TRPV1 negative (Figure 4(e)). TRPV4 expression in all samples of high grade carcinoma was similar to TRPV4 expression in normal urothelium as well as in low grade carcinoma (Figure 1). Immunolabeling was negative or weak in all cell layers of all high grade carcinoma samples (Figure 4(f)). Positive TRPV4 immunolabeling was detected in the lamina propria.

## 4. Discussion

**Figure 5:**
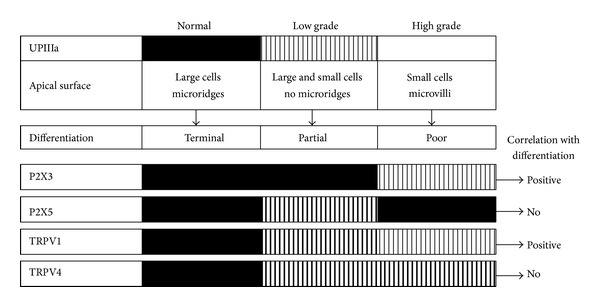
Summarised results of immunofluorescence, Western blotting, and scanning electron microscopy in normal human urothelium and in low and high grade papillary urothelial carcinomas. Uroplakin UPIIIa expression and apical surface appearance indicate differentiation stages of urothelial cells. P2X3, P2X5, TRPV1, and TRPV4 expressions are illustrated and their correlations with differentiation stage of urothelial cells are presented. Black squares indicate high protein expression, dashed squares denote moderate protein expression, and white squares represent no protein expression.

Unique differentiation of normal urothelium has been investigated since the 1970s, while its sensory role has been discovered only recently [7, 8]. Among sensory proteins, members of purinergic P2X receptors and transient receptor potential vanilloid (TRPV) channels are under intense investigation. The majority of studies have been conducted on animal tissues or cell culture models, while very little is known about their distribution and function in normal human urothelium or in urothelial tumours. We investigated the correlation between urothelial differentiation and sensory function-related proteins P2X3, P2X5, TRPV1, and TRPV4 in normal human urothelium and in low and high grade papillary urothelial carcinomas. All our results are summarised in Figure 5.

First we evaluated the differentiation of normal urothelium and that of low and high grade carcinomas. Two well-established criteria were used to determine urothelial cell differentiation. One is the expression and localization of uroplakins and the second criterion is the appearance of urothelial apical surface. In normal urothelium, there was strong expression of uroplakins, which were localized in superficial urothelial cell layer. Apical surface was scalloped, with microridges covering the umbrella cells. These characteristics demonstrate terminal differentiation of the urothelium, as proposed previously [21]. In low grade carcinoma decrease in uroplakin expression was detected and altered urothelial apical surface appearance was observed. Both criteria provide evidence for partial differentiation of low grade carcinoma [19, 21]. In high grade carcinoma there was no expression of uroplakins and superficial cells, which were covered with microvilli, were small and polymorphic. All these features point to poor differentiation of high grade carcinoma. Decreased urothelial differentiation is usually associated with incomplete barrier formation and bladder dysfunction. Since urothelial carcinoma may cause LUTS, we hypothesised that this involves also changes of some sensory proteins expression and localization.

Since the first demonstration of the P2X3 receptor in the human urothelium [31], only few studies confirmed the presence of P2X3 protein [32], while expression analyses revealed no P2X3 mRNA in the human urothelium [33]. These could be assigned to factors such as translational regulation, mRNA stability, and half-life of a protein [34]. In the present study, P2X3 immunoreactivity was observed throughout all urothelial cell layers of normal urothelium. Umbrella cells, which were terminally differentiated, were labelled stronger than cells in other layers, which is in coincidence with previous results [31]. Since differentiation of the urothelium progresses from basal cells to umbrella cells, it seems that P2X3 expression is related to cell differentiation stage. In low grade carcinoma we confirmed the expression of P2X3. Moreover, the expression and localization of P2X3 in partially differentiated low grade carcinoma, where some terminally differentiated superficial cells were preserved, were similar as in normal urothelium. In high grade carcinoma P2X3 expression was decreased when compared to normal urothelium and low grade carcinoma. Since high grade carcinoma was poorly differentiated, these results confirmed our abovementioned assumption that positive correlation exists between the expression of P2X3 and urothelial differentiation.

The expression of P2X5 in normal urothelium was confirmed, which is in coincidence with other reports [34]. The majority of low grade carcinoma samples exhibited no P2X5 expression, while only few showed positive immunolabeling in the superficial urothelial cells. In poorly differentiated high grade carcinoma strong expression of P2X5 in all cell layers was detected. Western blotting revealed similar level of P2X5 expression in the protein samples of highly differentiated normal urothelium and of poorly differentiated high grade carcinoma. It was shown that ATP significantly reduced cell proliferation in high grade bladder cancer and pharmacological profiling implicated P2X5 receptor in this antineoplastic response [28]. To our knowledge we showed for the first time that P2X5 receptors are expressed by the high grade papillary urothelial carcinoma.

It is known that bladder distension causes urothelial ATP release, which can directly depolarize and initiate firing in sensory nerves by activating P2X receptors [27]. Sensation and micturition are complex and not yet fully understood processes, which are altered during progression of diverse bladder diseases and related to various LUTS. Therefore, we suppose that decreased expression of P2X3 and increased expression of P2X5 detected in high grade carcinoma might be involved in pathogenesis and LUTS manifestations of this kind of carcinoma.

The remarkable finding that TRPV1 is not only expressed by afferent nerves but also in the urothelium [35, 36] gave rise to intensive studies of its expression and localization. Our results showed TRPV1 expression in normal urothelium with terminally differentiated umbrella cells. TRPV1 was not restricted to umbrella cells, as reported previously [29]. Moreover, downregulation of TRPV1 in urothelial cancers of human bladder was determined [17, 29] and the hypothesis that TRPV1 is involved in differentiation was postulated [16]. Our results support this hypothesis, since in partially differentiated low grade and in poorly differentiated high grade carcinoma its expression was decreased and abolished, respectively.

Several studies localized TRPV4 throughout all urothelial cell layers of normal urothelium [18, 37, 38]. Our results confirmed this data and showed variability among the levels of TRPV4 expression in the normal urothelium. We could not find any pattern in these findings that would point to any correlation between urothelial differentiation and TRPV4 channels. To our knowledge, there are no data about TRPV4 expression in urinary bladder tumours. Although Western blotting showed no variations in the expression of TRPV4 in normal urothelium and in low and high grade carcinomas, immunofluorescence revealed great diversity among different parts of the carcinomas. Some parts exhibited strong TRPV4 immunolabeling, while in others immunolabeling was weak and even parts with negative reaction were detected. It seems that TRPV4 was not correlated with urothelial differentiation and further research is necessary to clarify its role in bladder carcinogenesis.

## 5. Conclusions

Our results show that P2X3 is in correlation with urothelial differentiation and might be involved in high grade papillary carcinoma pathogenesis. We also confirm the correlation of TRPV1 with urothelial differentiation stage. Moreover, our study supports previous proposal that TRPV1 receptor should be accepted as a negative prognostic factor in patients with urothelial carcinoma. Regarding P2X5 and TRPV4 no direct correlation between their expression and urothelial differentiation is demonstrated. Nevertheless, new aspects concerning their localization variability in urothelial papillary carcinoma emerged indicating that they have a role in bladder functioning during pathogenesis.
